# Replacement of Lost Substance P Reduces Fibrosis in the Diabetic Heart by Preventing Adverse Fibroblast and Macrophage Phenotype Changes

**DOI:** 10.3390/cells10102659

**Published:** 2021-10-05

**Authors:** Alexander Widiapradja, Ainsley O. Kasparian, Samuel L. McCaffrey, Lauren L. Kolb, John D. Imig, Jessica L. Lacey, Giselle C. Melendez, Scott P. Levick

**Affiliations:** 1 Kolling Institute, St Leonards, NSW 2065, Australia; alexander.widiapradja@sydney.edu.au (A.W.); Ainsley.kasparian@sydney.edu.au (A.O.K.); sam.mccaffrey15@gmail.com (S.L.M.); 2 Faculty of Medicine and Health, The University of Sydney, Camperdown, NSW 2006, Australia; 3 School of Life Sciences, University of Technology Sydney, Ultimo, NSW 2007, Australia; 4 Drug Discovery Center, Medical College of Wisconsin, Milwaukee, WI 53226, USA; lkolb@mcw.edu (L.L.K.); jdimig@mcw.edu (J.D.I.); 5 Section on Cardiology, Department of Internal Medicine, Wake Forest School of Medicine, Winston-Salem, NC 27157, USA; jmccanle@wakehealth.edu (J.L.L.); gmelende@wakehealth.edu (G.C.M.); 6 Section on Comparative Medicine, Department of Pathology, Wake Forest School of Medicine, Winston-Salem, NC 27157, USA

**Keywords:** diabetes, cardiomyopathy, heart, neuropeptide, inflammation, myofibroblast

## Abstract

Reduced levels of the sensory nerve neuropeptide substance P (SP) have been reported in the diabetic rat heart, the consequence being a loss of cardioprotection in response to ischemic post-conditioning. We considered whether this loss of SP also predisposes the heart to non-ischemic diabetic cardiomyopathy in the form of fibrosis and hypertrophy. We report that diabetic Lepr^db/db^ mice have reduced serum SP and that administration of exogenous replacement SP ameliorated cardiac fibrosis. Cardiac hypertrophy did not occur in Lepr^db/db^ mice. Cardiac fibroblasts exposed to high glucose converted to a myofibroblast phenotype and produced excess extracellular matrix proteins; this was prevented by the presence of SP in the culture media. Cardiac fibroblasts exposed to high glucose produced increased amounts of the receptor for advanced glycation end products, reactive oxygen species and inflammatory cytokines, all of which were prevented by SP. Cultured macrophages assumed an M1 pro-inflammatory phenotype in response to high glucose as indicated by increased TNF-α, CCL2, and IL-6. SP promoted a shift to the reparative M2 macrophage phenotype characterized by arginase-1 and IL-10. Lepr^db/db^ mice showed increased left ventricular M1 phenotype macrophages and an increase in the M1/M2 ratio. Replacement SP in Lepr^db/db^ mice restored a favorable M1 to M2 balance. Together these findings indicate that a loss of SP predisposes the diabetic heart to developing fibrosis. The anti-fibrotic actions of replacement SP involve direct effects on cardiac fibroblasts and macrophages to oppose adverse phenotype changes. This study identifies the potential of replacement SP to treat diabetic cardiomyopathy.

## 1. Introduction

Patients with Type 2 diabetes mellitus (T2DM) have a 20–40% higher incidence of heart failure [1] and a 33% greater risk of hospitalization [2]. While in many instances the pathogenesis of T2DM-associated heart failure may be due to hypertension and coronary artery disease, evidence indicates that a specific diabetic cardiomyopathy, characterized by cardiomyocyte hypertrophy, microvascular damage and interstitial cardiac fibrosis, can occur independent of comorbidities [3,4,5,6]. This cardiomyopathy, particularly fibrosis, leads to impaired left ventricular (LV) compliance and diastolic dysfunction, ultimately manifesting as heart failure with preserved ejection fraction (HFpEF) [7]. Despite the increased morbidity and mortality associated with T2DM-induced cardiac fibrosis [8], there are no effective treatment strategies to ameliorate or reverse this fibrosis.

An accumulating body of evidence has demonstrated that the neuropeptide substance P (SP) plays a critical role in regulating remodeling of the cardiac extracellular matrix (ECM) [9,10,11]. Encoded by the *Tac1* gene, SP belongs to a family of peptides known as tachykinins, which are expressed predominantly in the nervous and immune systems. SP has complex actions in the heart, dependent on disease etiology and time-course [9]. For example, myocarditis [12,13,14], magnesium-deficiency [15], chemotherapy toxicity [16,17], and hypertension [10] all increase SP levels to promote fibrosis and hypertrophy. Conversely, increased SP acutely following ischemia provides protection against cardiomyocyte death and reduces infarct size [18,19]. In contrast, T2DM causes an overall reduction in neuropeptides, including SP [20,21]. In the diabetic rat heart, SP was reduced concomitant with a loss of cardioprotection typically imparted by post-conditioning [22]. Post-conditioning cardioprotection was restored by replacement of SP [22]. With this in mind, we considered whether the loss of SP in diabetes also predisposes the heart to developing non-ischemic diabetic cardiomyopathy, particularly fibrosis. Accordingly, in this study we investigated: (1) the extent to which there is a causal relationship between the loss of SP and cardiac fibrosis using a mouse model of T2DM; (2) the extent to which SP opposes high glucose-induced changes to cardiac fibroblast phenotype and function; and (3) the extent to which SP influences macrophage phenotype and function under high glucose conditions.

## 2. Materials and Methods

### 2.1. Experimental Design

Mice were housed under standard environmental conditions and were maintained on LabDiet5K52 NIH commercial mouse chow and tap water ad libitum. Studies to establish causation between SP level and myocardial outcomes were performed using the Lepr^db/db^ (B6.BKS(D)-Lepr*^db^*/J) mouse model of diabetes and obesity. Twelve-week-old male Lepr^db/db^ mice were randomly assigned to one of two groups: 1) Lepr^db/db^ mice + saline (*n* = 10); or 2) Lepr^db/db^ mice + SP (50 μg/day, s.c. injection, *n* = 10). This dose of SP was determined from a group of pilot mice that showed reduced fibrosis with no obvious side effects. Age-matched male C57BL/6 mice (*n* = 6) served as non-diabetic controls. All mice were purchased from Jackson Laboratories (Bar Harbor, ME, USA). Saline or SP were administered daily for 4 weeks, so that at the end of the experimental period mice were 16 weeks of age. At the experimental endpoint, systolic blood pressure was assessed by tail cuff method in unanesthetized animals that had been acclimatized to the apparatus. Non-fasting blood glucose was measured by sampling blood from the tail vein prior to euthanasia in anaesthetized mice (2% inhaled isoflurane). Proper analgesia for euthanasia was evaluated by palpebral reflex, toe pinch reflex, and corneal reflex. Euthanasia was accomplished by removal of the heart. The right ventricle (RV) was then separated from the LV plus septum and both were weighed. The apical portion of the LV was snap frozen in liquid nitrogen and stored at −80 °C, while the mid-papillary portion was fixed in zinc formalin for histological analysis. The lungs were also removed, blotted and weighed. These studies conformed to the principles of the National Institutes of Health Guide for the Care and Use of Laboratory Animals, and the protocols were approved by the Institutional Animal Ethics Committee under the approval reference number RESP17/254.

### 2.2. Collagen Volume Fraction

Cardiac fibrosis was identified by assessing collagen volume fraction by staining of fibrillar collagen with picrosirius red stain. Formalin fixed LV sections of 5 μm thickness were mounted onto slides and underwent rehydration before incubation in phosphomolybdic acid (0.2%) and then staining with picrosirius red (0.1% Sirius Red F3BA in picric acid). All slides then underwent dehydration before being mounted and cover slipped with DEPEX. Collagen volume fraction was quantified as we have previously described [10,11,17,23,24,25]. Briefly, 10 images per LV section were acquired using a 20x objective and analyzed with Image J software. Perivascular areas were excluded from the analysis.

### 2.3. Collagen Hybridizing Peptide Staining

Formalin fixed LV sections of 5 μm thickness underwent rehydration before they were stained with 5-FAM conjugated collagen hybridizing peptide (10 μM, 3Helix) overnight at 4 °C. The slides were then washed with PBS and cover slipped using Prolong Diamond containing DAPI nuclear stain (Life Technologies). A positive control was included by incubating an LV section in hot water to purposely denature the collagen prior to staining overnight. The images were acquired using confocal microscope (Leica, Buffalo Grove, IL, USA) at 20× objective.

### 2.4. Cardiomyocyte Cross-Sectional Area

Formalin fixed LV sections of 5 μm thickness underwent rehydration before staining with fluorescent (488)-tagged wheat germ agglutinin (WGA) (20 μg/mL, Life Technologies) for visualization of cell membranes, which allowed the discrimination of cardiomyocytes vs. non-cardiomyocytes. LV sections were then cover slipped with Prolong Diamond containing DAPI stain (Life Technologies, Carlsbad, CA, USA) to visualize the presence of nuclei. Quantification of the cell size was performed with inForm^®^ image analysis software. Five fields from each LV section were imaged and analyzed. Only cells with well-defined cell membranes, visible cell nuclei, and a circular shape in the short-axis were selected as cardiomyocytes. Each field was analyzed using the software after defining cardiomyocytes. The mean cardiomyocyte cross-sectional area was calculated for each mouse LV section.

### 2.5. Isolation and Treatment of Cardiac Fibroblasts

Cardiac fibroblasts were isolated from the LV of male C57BL/6 mice (8 weeks of age) similarly to how we have described previously [10,24,25,26,27]. Briefly, the heart was removed from mice anesthetized with continuously inhaled isoflurane (2%). The LV and septum were separated from the RV and minced and digested by a series of 5 incubations with 100 ng/µL Liberase TM (Roche) at 37 °C for 15 min. The cell pellets were resuspended in DMEM-F12 media supplemented with 10% FBS and plated. Non-adherent cells were removed by washing. Fibroblasts were used after only one passage to minimize changes in phenotype associated with culture. Before treatment, all fibroblasts were serum-starved in DMEM-F12 media for 24 h on gelatin-coated 6-well plates, and then treated with either normal glucose (5 mM), high glucose (25 mM), or high glucose media containing SP at 10, 30, 100, 300, and 1000 nM for 24 h. All treatments were performed in DMEM-F12 containing 1.5% FBS. Human cardiac fibroblasts were purchased from Sigma-Aldrich (306-05A, St Louis, MO, USA). Before high glucose treatment, the fibroblasts were serum-starved in DMEM media for 24 hrs on gelatin-coated 6-well plates, and then treated with normal glucose (5 mM), high glucose (25 mM), or high glucose containing SP at 10, 30, 100, 300, and 1000 nM for 24 h. All treatments were performed in DMEM containing 1.5% FBS. The cells were used at passage 2.

### 2.6. Bone Marrow-Derived Macrophages (BMMΦ)

Bone marrow cells were extracted from the tibia and femur bones of 6 to 8-week-old male WT mice and centrifuged at 800 rpm for 8 min in DMEM supplemented with 1% penicillin/streptomycin and 10% FBS. The pellet was then resuspended in DMEM containing 10 ng/mL of colony stimulating factor (Sigma-Aldrich, St Louis, MO, USA) and the cells plated in flasks for population expansion. Ninety percent pure populations of BMMΦ’s were obtained after 7 to 10 days. For in vitro experiments, BMMΦ’s were treated with media containing either normal glucose (5 mM), high glucose (25 mM), or high glucose containing SP at 10, 30, 100, 300, and 1000 nM for 24 h.

### 2.7. Cell Proliferation

Cell proliferation was determined using the CyQuant Proliferation Assay Kit (Life Technologies, Carlsbad, CA, USA) according to the manufacturer’s protocol. Isolated mouse cardiac fibroblasts were cultured in 96 well plates at 1 × 10^4^ cells/well for 24 h and treated with normal glucose (5 mM), high glucose (25 mM), or high glucose media containing SP at 10, 30, 100, 300, and 1000 nM for 24 h. The assay was performed in duplicate and read using a fluorescence microplate reader at an excitation wavelength of 485 nm.

### 2.8. Cell Migration

Migration was assessed using transwell membranes. Briefly, WT mouse cardiac fibroblasts were seeded on transwell membranes at 2 × 10^4^ cell density. The media in the well was either normal glucose (5 mM), high glucose (25 mM) or high glucose containing SP at 100 or 1000 nM. Fibroblasts were allowed to migrate through the porous upper-level membrane for 24 h. At the end of 24 h, the membranes were fixed in methanol, cut and mounted with Prolong Diamond containing DAPI stain (Life Technologies, Carlsbad, CA, USA) to identify the migrated cells on the underside of the membrane, which were then counted.

### 2.9. Immunolabeling

Formalin fixed LV sections of 5 μm thickness underwent rehydration before incubation in a pressure cooker with Tris-EDTA pH 9 (Dako) for antigen retrieval. The sections were labelled with anti-Mac-2 (1:100, Cedarlane, Burlington, Canada) and anti-CD86 (1:100, Santa Cruz, Dallas, TX, USA) to identify macrophages of the pro-inflammatory M1 phenotype, following blocking for non-specific binding. Mac-2 labelling was visualized using Alexa 568-goat anti rat secondary antibody (1:100, LifeTechnologies, Carlsbad, CA, USA) and CD86 was visualized using Alexa 488-goat anti rabbit secondary antibody (1:100, LifeTechnologies, Carlsbad, CA, USA). M1 macrophages were considered as those cells that labeled positive for both Mac-2 and CD86. Macrophages of the M2 phenotype were those cells that labeled positive to anti-CD206 (1:100, Abcam, Cambridge, United Kingdom) after visualization with Alexa 488-goat anti rabbit secondary antibody (1:100, LifeTechnologies, Carlsbad, CA, USA). All sections were also labeled with DAPI (Sigma-Aldrich, St Louis, MO, USA) to identify nuclei and cover-slipped using VectaShield (Vector, Burlingame, CA, USA). M1 and M2 positive macrophages were visualized and counted using a fluorescence microscope. Representative images were taken using a confocal microscope (Leica, Buffalo Grove, IL, USA). The quantification of M1 and M2 was represented both as individual count and as a ratio of M1 to M2. The M1/M2 ratio was expressed as a percentage for each cell type, with the total of both equaling 100%.

### 2.10. Hydroxyproline Assay

Total collagen released by cultured human cardiac fibroblasts (Sigma-Aldrich, 306-05A, St Louis, MO, USA) was assessed by measuring hydroxyproline levels in the culture media. Briefly, 100 µL of fibroblast cell culture media was incubated with 100 µL of 6N HCl and hydrolysed at 107 °C for 18 h. The samples were dried in a vacuum centrifuge and then reconstituted with 500 μL of dH_2_O. They were then oxidized with 250 μL of chloramine T reagent (Sigma-Aldrich, St Louis, MO, USA) and developed with 250 μL of Ehrlich’s reagent (Sigma-Aldrich, St Louis, MO, USA). Then, 200 μL of each sample, including the standards was aliquoted into 96 well plates before being read at absorbance wavelength of 550 nm. All samples were run in duplicate and averaged with hydroxyproline level determined from a hydroxyproline standard curve.

### 2.11. ELISA Protein Measurement

A commercially available ELISA kit was used to determine SP serum levels for mice (Cayman Chemicals, Ann Arbor, MI, USA). Collagen Iα1 (Novus Biologicals, Littleton, CO, USA), collagen IIIα1 (Novus Biologicals, Littleton, CO, USA), fibronectin (MyBioSource, San Diego, CA, USA), and laminin (Abcam, Cambridge, United Kingdom) were measured in the media to assess fibroblast production of ECM proteins. Alpha-smooth muscle actin (α-SMA, Novus Biologicals, Littleton, CO, USA) and the receptor for advanced glycation end products (RAGE, MyBioSource, San Diego, CA, USA) were assessed in cardiac fibroblast lysates. Lysyl oxidase (LOX, MyBioSource, San Diego, CA, USA), bone morphogenic protein 1 (BMP-1, Novus Biologicals, Littleton, CO, USA), soluble RAGE (sRAGE, MyBioSource, San Diego, CA, USA), hydrogen peroxide (H_2_O_2,_ Cell BioLabs Inc, San Diego, CA, USA), superoxide dismutase (Cell BioLabs Inc, San Diego, CA, USA), and nitrate/nitrite (nitric oxide, NO, Cell BioLabs Inc, San Diego, CA, USA) were measured in fibroblast media. TNF-α, CCL2, IL-4, IL-6, and IL-10 were also assessed in fibroblast media by commercial ELISA (BD Biosciences, Franklin Lakes, NJ, USA). TNF-α, CCL2, and IL-6 (BD Biosciences, Franklin Lakes, NJ, USA) were assessed in macrophage media as markers of the M1 macrophage phenotype. IL-10 (BD Biosciences, Franklin Lakes, NJ, USA) assessed in the macrophage media, and arginase-1 (MyBioSource, San Diego, CA, USA) assessed in macrophage lysates were measured as markers of the M2 macrophage phenotype. S100A9 (MyBioSource, San Diego, CA, USA) was measured in macrophage media as a marker of AGE release by macrophages. All samples were run in duplicate and averaged.

### 2.12. Statistical Analysis

All grouped data were expressed as mean ± SD or SEM as appropriate. Grouped data comparisons were made by *t*-test or one-way ANOVA for comparison of three or more groups. When ANOVA analysis identified a significant overall effect, intergroup comparisons were made using the Tukey post hoc test. Statistical significance was taken at *p* < 0.05. Pearson’s correlation was used to investigate the relationship between macrophage phenotype and cardiac fibrosis. Values for all groups were combined. Statistical significance of the correlation co-efficient was taken at *p* < 0.05.

## 3. Results

### 3.1. Biometrics

**Figure 1:**
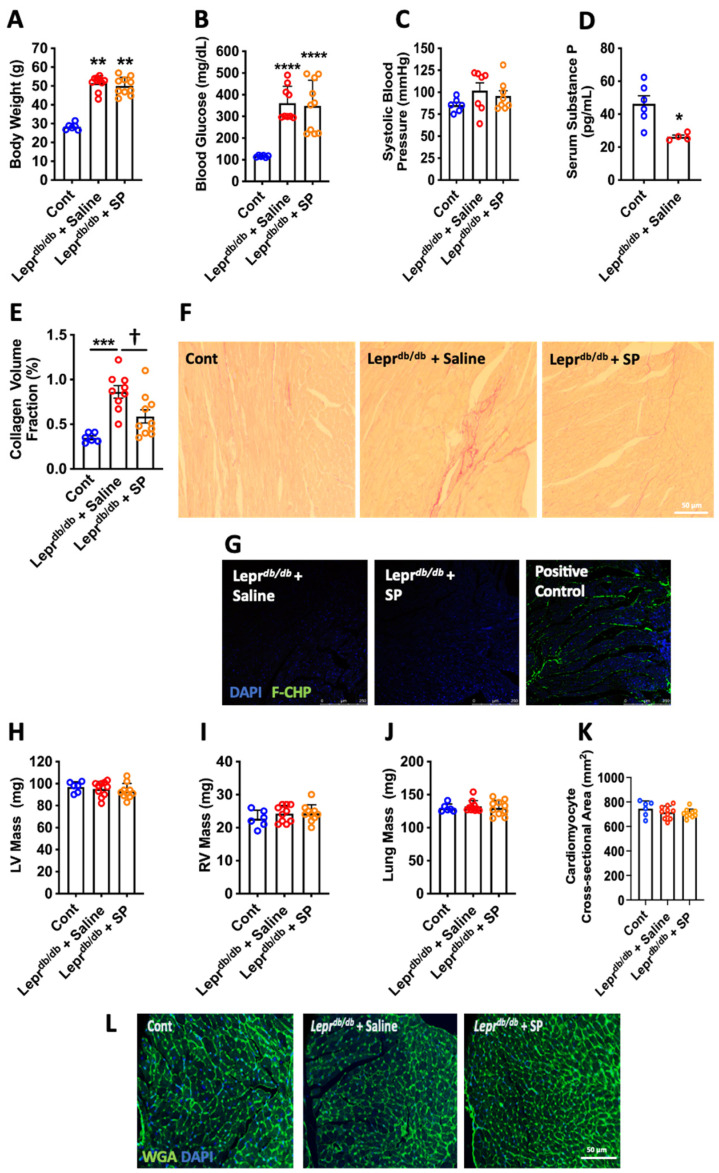
Lepr^db/db^ mouse biometrics. (**A**) Body weight; (**B**) blood glucose; and (**C**) systolic blood pressure in control (Cont, *n* = 6), Lepr^db/db^ + saline (*n* = 10), and Lepr^db/db^ + SP mice (*n* = 10); (**D**) serum SP levels in Cont (*n* = 6) and Lepr^db/db^ + saline mice (*n* = 4). Data are expressed as mean ± SD and were analyzed by one-way ANOVA with Tukey post hoc test, except for serum SP levels, which are expressed as mean ± SEM and were analyzed by unpaired t-test. * *p* < 0.05 vs. Cont, ** *p* < 0.01 vs. Cont, **** *p* < 0.0001 vs. Cont. Replacement SP ameliorates cardiac fibrosis. (**E**) Collagen volume fraction quantification; and (**F**) representative images of picrosirious red staining for Cont (*n* = 6), Lepr^db/db^ + saline (*n* = 10), and Lepr^db/db^ + SP mice (*n* = 10). Data are expressed as mean ± SEM and were analyzed by one-way ANOVA with Tukey post hoc test; *** *p* < 0.001 vs. Cont, † *p* < 0.05 vs. Lepr^db/db^ + saline. (**G**) Representative images of unfolded collagen as identified by FAM-conjugated collagen hybridizing peptide (F-CHP) labeling of LV sections. Positive Control = LV section boiled in H_2_O to damage collagen prior to labeling with collagen hybridizing peptide (green fluorescence = unfolded collagen, Blue = DAPI). Replacement SP does not alter organ hypertrophy. (**H**) LV mass; (**I**) RV mass; and (**J**) Lung mass; (**K**) cardiomyocyte cross-sectional area; and (**L**) representative images of wheat germ agglutinin (WGA) staining for Cont (*n* = 6), Lepr^db/db^ + saline (*n* = 10), and Lepr^db/db^ + SP mice (*n* = 10). Data are expressed as mean ± SD and were analyzed by one-way ANOVA with Tukey post hoc test. (WGA = green, DAPI = blue).

Lepr^db/db^ mice administered either saline or SP exhibited significantly greater body weights (*p* < 0.01, Figure 1A) and blood glucose levels (*p* < 0.0001, Figure 1B) than non-diabetic control mice. There were no statistical differences for systolic blood pressure between any of the groups (Figure 1C). Lepr^db/db^ mice had significantly reduced serum SP levels compared to control mice (*p* < 0.05, Figure 1D).

### 3.2. Replacement SP Reduces Cardiac Fibrosis in Lepr^db/db^ Mice

Increased LV collagen volume fraction was observed in Lepr^db/db^ mice compared to controls (*p* < 0.001, Figure 1E,F), indicative of fibrosis. Replacement SP significantly reduced fibrosis compared to the Lepr^db/db^ + saline group (*p* < 0.05) and was not significantly different from controls (*p* = 0.07). A collagen hybridizing peptide was used to identify damaged unfolded collagen. While the heat-treated positive control LV section showed extensive damaged collagen, there was no damaged collagen identified in LV sections from Lepr^db/db^ + saline or Lepr^db/db^ + SP mice (Figure 1G).

### 3.3. Organ Hypertrophy

There were no statistical differences for LV mass, RV mass, or lung mass between any of the groups (Figure 1H–J). Similarly, WGA staining identified no differences in cardiomyocyte cross-sectional area between groups (Figure 1K,L).

### 3.4. SP Opposes the High Glucose-Induced Pro-Fibrotic Phenotype in Cardiac Fibroblasts

**Figure 2:**
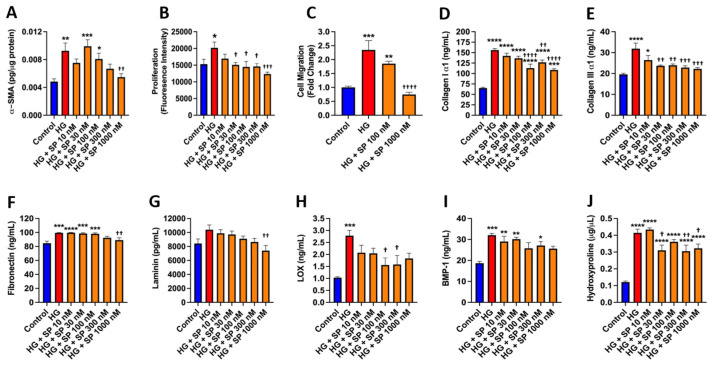
SP opposes the effects of high glucose on cardiac fibroblast phenotype and function. Isolated mouse cardiac fibroblast (**A**) α-smooth muscle actin (α-SMA) in cell lysate; (**B**) cellular proliferation; (**C**) and cell migration in response to normal glucose (Control, 5 mM), high glucose (HG, 25 mM), and HG with increasing concentrations of SP (10 to 1000 nM). (**D**) collagen I α1; (**E**) collagen III α1; (**F**) fibronectin; (**G**) laminin; (**H**) lysyl oxidase (LOX); and (**I**) bone morphogenic protein-1 (BMP-1) measured in the cell culture media from isolated cardiac fibroblasts in response to control, HG, and HG with increasing concentrations of SP (10 to 1000 nM). (**J**) hydroxyproline measured in the media from cultured human cardiac fibroblasts in response to control, HG, and HG with increasing concentrations of SP (10 to 1000 nM). Data are expressed as mean ± SEM and were analyzed by one-way ANOVA with Tukey post hoc test; * *p* < 0.05 vs. control, ** *p* < 0.01 vs. control, *** *p* < 0.001 vs. control, **** *p* < 0.0001 vs. control, † *p* < 0.05 vs. HG, †† *p* < 0.01 vs. HG, ††† *p* < 0.001 vs. HG, †††† *p* < 0.0001 vs. HG. *n* = 4–6 for control and *n* = 5–6 for all other groups.

Mouse cardiac fibroblast α-SMA, proliferation, and migration were assessed as indicators of myofibroblast conversion (Figure 2A–C). There were significant increases in α-SMA, proliferation, and migration in response to high glucose compared to controls (*p* < 0.01, *p* < 0.05 and *p* < 0.001, respectively), indicative of increased myofibroblast conversion.

Treatment with SP induced a concentration-dependent decrease in α-SMA that reached statistical significance at 1000 nM (*p* < 0.01 vs. high glucose, Figure 2A). Fibroblast proliferation was prevented by SP treatment at concentrations of 30 nM (*p* < 0.05) to 1000 nM (*p* < 0.001, Figure 2B), while SP at a concentration of 1000 nM also prevented increases in migration (*p* < 0.0001 vs. high glucose, Figure 2C). We then assessed ECM proteins released into the media as additional markers of a pro-fibrotic fibroblast phenotype. High glucose promoted increased collagen Iα1 release in comparison to normal glucose conditions (*p* < 0.0001, Figure 2D). Treatment of high glucose exposed fibroblasts with SP significantly attenuated collagen Iα1 production at 100 nM (*p* < 0.0001 vs. high glucose), 300 nM (*p* < 0.01 vs. high glucose), and 1000 nM (*p* < 0.0001 vs. high glucose). High glucose likewise promoted increased collagen IIIα1 production in comparison to normal glucose conditions (*p* < 0.0001, Figure 2E). SP significantly attenuated high glucose-induced collagen IIIα1 production at 30 nM (*p* < 0.01 vs. high glucose), 100 nM (*p* < 0.01 vs. high glucose), 300 nM (*p* < 0.001 vs. high glucose), and 1000 nM (*p* < 0.001 vs. high glucose). Fibronectin release was also significantly increased in response to high glucose (*p* < 0.001, Figure 2F). SP at the 1000 nM concentration significantly reduced fibronectin production (*p* < 0.01 vs. high glucose). While not statistically significant, laminin tended to be increased by high glucose (Figure 2G). SP at 1000 nM reduced laminin to below high glucose levels (*p* < 0.01). Interestingly, there was a significant increase in the collagen cross-linking enzyme LOX in response to high glucose (*p* < 0.001, Figure 2H), which was significantly reduced by SP (*p* < 0.05 vs. high glucose). BMP-1 is a key enzyme in the process of mature collagen formation as it removes the C-terminal portion of the pro-collagen molecule. BMP-1 was significantly increased in response to high glucose (*p* < 0.001, Figure 2I). Although there was no statistically significant reduction in BMP-1 in response to any SP concentration, there was a trend for a concentration-dependent decrease with SP. We then examined the translational relevancy of our fibroblast phenotype findings by assessing hydroxyproline as a marker of total collagen release by cultured human cardiac fibroblasts. High glucose promoted an increased hydroxyproline concentration in comparison to normal glucose conditions (*p* < 0.0001, Figure 2J). Treatment of high glucose exposed human fibroblasts with SP significantly attenuated hydroxyproline concentration at 30 nM (*p* < 0.05 vs. high glucose), 300 nM (*p* < 0.01 vs. high glucose), and 1000 nM (*p* < 0.05 vs. high glucose).

### 3.5. SP Reduces RAGE and Opposes High Glucose-Induced Oxidative Stress in Cardiac Fibroblasts

**Figure 3:**
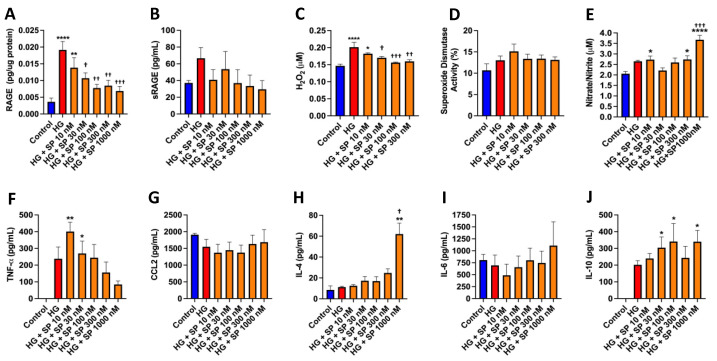
SP reduces RAGE and oxidative stress while increasing nitric oxide in isolated cardiac fibroblasts in response to high glucose. (**A**) Receptor for advanced glycation end product (RAGE) in cardiac fibroblast lysates; (**B**) soluble RAGE (sRAGE); and (**C**) hydrogen peroxide (H_2_O_2_) in the culture media of isolated cardiac fibroblasts in response to normal glucose (control, 5 mM), high glucose (HG, 25 mM), and HG with increasing concentrations of SP (10 to 1000 nM); (**D**) superoxide dismutase levels in cardiac fibroblast lysates in response to control, HG, and HG with increasing concentrations of SP; (**E**) total nitrate/nitrite as a marker of nitric oxide (NO) production in the culture media of isolated cardiac fibroblasts in response to control, HG, and HG with increasing concentrations of SP. SP alters cytokine production by isolated cardiac fibroblasts in response to high glucose. (**F**) TNF-α, (**G**) CCL2, (**H**) IL-4, (**I**) IL-6, and (**J**) IL-10 levels in isolated cardiac fibroblast cell culture media in response to normal glucose (control, 5 mM), high glucose (HG, 25 mM), and HG with increasing concentrations of SP (10 to 1000 nM). Data are expressed as mean ± SEM and were analyzed by one-way ANOVA with Tukey post hoc test; * *p* < 0.05 vs. control, ** *p* < 0.01 vs. control, **** *p* < 0.0001 vs. control, † *p* < 0.05 vs. HG, †† *p* < 0.01 vs. HG, ††† *p* < 0.001 vs. HG. *n* = 4–6 for control and *n* = 5–6 for all other groups.

RAGE mediates many of the adverse effects of high glucose and was significantly upregulated in response to high glucose in cell lysates from isolated mouse cardiac fibroblasts (*p* < 0.0001, Figure 3A). SP concentration-dependently reduced RAGE, with a maximal effect at 1000 nM (*p* < 0.001 vs. high glucose). sRAGE, which acts as a scavenger for advanced glycation end products (AGE’s), was non-significantly increased in response to high glucose and tended to be decreased by SP (Figure 3B). H_2_O_2_ was assessed in the media from cultured cardiac fibroblasts as a marker of oxidative stress. High glucose promoted increased extracellular H_2_O_2_ in comparison to normal glucose conditions (*p* < 0.0001, Figure 3C). Treatment of high glucose fibroblasts with SP at 30 nM (*p* < 0.05), 100 nM (*p* < 0.001), and 300 nM (*p* < 0.01) prevented the increase in released H_2_O_2_. There were no significant changes in activity level of the antioxidant superoxide dismutase in any of the groups (Figure 3D). Total nitrate/nitrite was assessed in the media of cardiac fibroblasts as a measure of NO production. There was a tendency for NO to be increased in response to high glucose and was statistically increased at 10 nM of SP (*p* < 0.05, vs. Control), and again at 300 nM (*p* < 0.05, vs. Control) and 1000 nM (*p* < 0.0001 vs. Control, *p* < 0.001 vs. high glucose, Figure 3E).

### 3.6. Effects of SP on Cytokine Production by Cardiac Fibroblasts in Response to High Glucose

Cardiac fibroblasts did not produce TNF-α under normal glucose conditions (Figure 3F). However, high glucose did promote TNF-α production. Treatment of fibroblasts exposed to high glucose with SP caused a concentration-dependent reduction in TNF-α. CCL2 was not significantly altered in response to high glucose or SP treatment (Figure 3G). IL-4 production was not altered in response to high glucose, however, SP did cause a concentration-dependent rise in IL-4, with a dramatic increase at SP 1000 nM that was significantly different to control (*p* < 0.01) and high glucose (*p* < 0.05, Figure 3H). IL-6 was not altered in response to high glucose or SP treatment (Figure 3I). Cardiac fibroblasts did not produce IL-10 under normal glucose conditions (Figure 3J), however, exposure to high glucose promoted IL-10 production. Treatment with SP caused a concentration-dependent increase in IL-10 compared to control (*p* < 0.05 at SP 30 nM, 100 nM, and 1000 nM).

### 3.7. SP Alters Macrophage Phenotype in Response to High Glucose In Vitro

**Figure 4:**
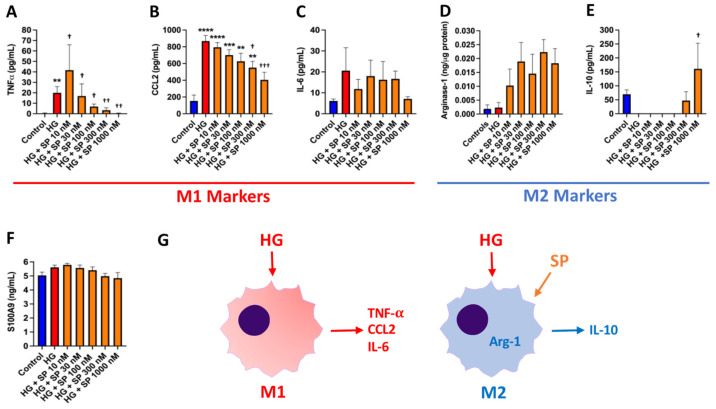
SP promotes an M2 macrophage phenotype in vitro. Macrophage M1 phenotype markers (**A**) TNF-α; (**B**) CCL2; and (**C**) IL-6 in cultured BMMF culture media in response to normal glucose (control, 5 mM), high glucose (HG, 25 mM), and HG with increasing concentrations of SP (10 to 1000 nM). M2 macrophage phenotype markers (**D**) arginase-1; and (**E**) IL-10 in cultured BMMF lysates and media, respectively, in response to normal glucose (control, 5 mM), high glucose (HG, 25 mM), and HG with increasing concentrations of SP (10 to 1000 nM); (**F**) S100A9 levels in cultured BMMF culture media in response to normal glucose, HG, and HG with increasing concentrations of SP; and (**G**) Schematic showing the M1 and M2 macrophage phenotype and markers in response to HG, as well as the effect of SP on macrophage phenotype. Data are expressed as mean ± SEM and were analyzed by one-way ANOVA with Tukey post hoc test; ** *p* < 0.01 vs. control, *** *p* < 0.001 vs. control, **** *p* < 0.0001 vs. control, † *p* < 0.05 vs. HG, †† *p* < 0.01 vs. HG, ††† *p* < 0.001 vs. HG. *n* = 4–6 for control and *n* = 5–6 for all other groups.

We utilized cultured BMMΦ’s to determine whether SP could oppose the changes in macrophage phenotype that occur in response to high glucose. We firstly examined cytokines indicative of a ‘pro-inflammatory’ M1 macrophage phenotype (TNF-α, CCL2, and IL-6). BMMΦ’s produced very little TNF-α under normal glucose conditions (Figure 4A). However, high glucose promoted a significant increase in TNF-α production (*p* < 0.01 vs. control), with SP treatment showing a concentration-response reduction in TNF-α (*p* < 0.01 vs. high glucose) that returned to control levels. CCL2 was dramatically increased in response to high glucose (*p* < 0.0001 vs. control, Figure 4B). Similar to TNF-α, SP induced a concentration-dependent decrease in CCL2 production that was significantly lower than high glucose alone at the 300 nM (*p* < 0.05 vs. high glucose) and 1000 nM (*p* < 0.001 vs. high glucose) concentrations. There was a non-significant trend for IL-6 to be increased by high glucose (Figure 4C). While SP did not significantly alter high glucose-induced IL-6 production, there was a strong trend for IL-6 to be lower in response to SP 1000 nM. We then examined markers of the ‘reparative’ M2 macrophage phenotype (arginase-1 and IL-10). There was no effect of high glucose on arginase-1 in BMMΦ cell lysates (Figure 4D), however, SP was able to dramatically increase arginase-1 production. High glucose caused a loss of IL-10 release by BMMΦ (Figure 4E). Treatment with SP at 300 nM and 1000 nM (*p* < 0.05 vs. high glucose) was able to restore IL-10 production. S100A9 is a pro-inflammatory mediator and AGE that is produced by macrophages. High glucose tended to increase S100A9 production, although this was not significant (Figure 4F). SP caused a concentration-dependent reduction in S100A9 release, although no concentration reached a statistically significant effect. Figure 4G depicts the induction of an M1 macrophage phenotype in response to high glucose, and the shift to an M2 phenotype with the presence of SP.

### 3.8. SP Alters Macrophage Phenotype in Response to High Glucose In Vivo

**Figure 5:**
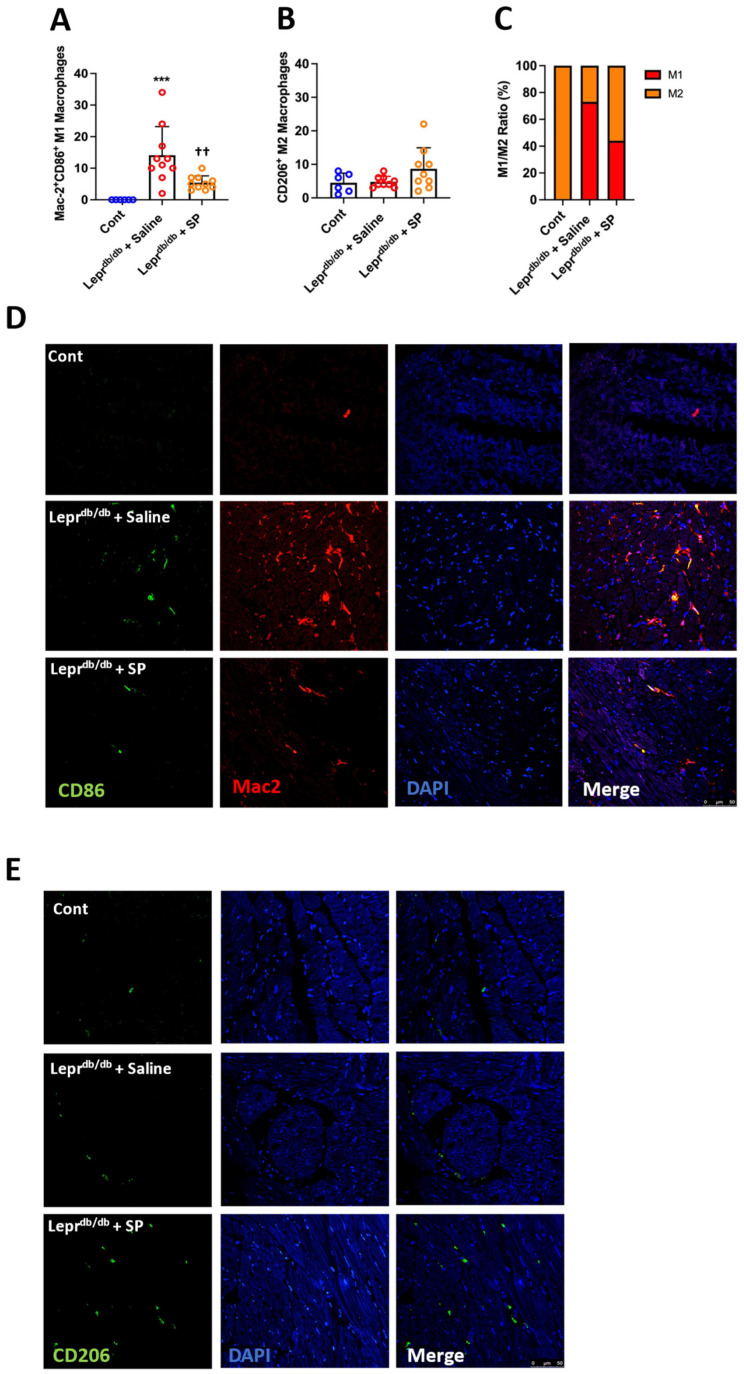
SP promotes a shift towards an M2 macrophage phenotype in vivo. (**A**) Number of M1 macrophages (Mac-2^+^CD86^+^) per LV section; (**B**) number of M2 macrophages (CD206^+^) per LV section; and (**C**) ratio of M1 to M2 macrophages per LV section for Cont (*n* = 6), Lepr^db/db^ + saline (*n* = 10), and Lepr^db/db^ + SP mice (*n* = 10). (**D**) Representative images for M1 macrophages (Mac-2^+^CD86^+^); and (**E**) Representative images for M2 macrophages (CD206^+^). Data are expressed as mean ± SD and were analyzed by one-way ANOVA with Tukey post hoc test. *** *p* < 0.001, †† *p* < 0.01.

M1 pro-inflammatory macrophages were identified in the heart by co-labeling with Mac-2 and CD86, while M2 reparative macrophages were identified by the presence of CD206. We did not identify any Mac-2^+^CD86^+^ M1 macrophages in control mice, indicating that macrophages in the normal mouse heart were not pro-inflammatory. However, untreated Lepr^db/db^ mice exhibited significantly increased numbers of Mac-2^+^CD86^+^ pro-inflammatory M1 macrophages compared to controls (*p* < 0.001, Figure 5A,D). Replacement SP significantly reduced the number of M1 macrophages (*p* < 0.01 vs. Lepr^db/db^ + saline) and was not significantly different from controls (*p* = 0.19). For CD206^+^ ‘reparative’ M2 macrophages, there were no statistically significant differences between any of the groups, although there did tend to be more M2 macrophages in Lepr^db/db^ + SP hearts (Figure 5B,E). When considering the ratio of M1 to M2 macrophages, control hearts were 100% M2 macrophage phenotype, whereas Lepr^db/db^ + saline hearts showed a dramatic shift to the M1 macrophage phenotype (73%, Figure 5C). Replacement SP promoted a shift in ratio whereby M2 macrophages again became the dominant phenotype at 54%.

**Figure 6:**
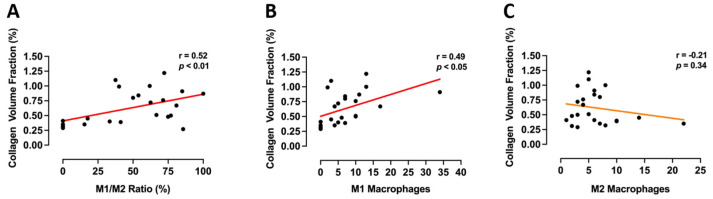
Macrophage phenotype associates with collagen volume fraction. Pearson’s correlations for (**A**) M1/M2 ratio; (**B**) M1 (Mac-2^+^CD86^+^) macrophages; and (**C**) M2 (CD206^+^) macrophages with collagen volume fraction in hearts from Cont, Lepr^db/db^ + saline, and Lepr^db/db^ + SP mice. Individual data points for all groups were included and analysed by Pearson correlation co-efficient.

When M1/M2 ratio was correlated to collagen volume fraction to determine the strength of the relationship between the two, there was a significant positive correlation (r = 0.52, *p* < 0.01) between a higher M1/M2 ratio and increased collagen volume fraction (Figure 6A). When separating parameters into M1 and M2 phenotype versus collagen volume fraction, there was a significant positive correlation between M1 macrophages and collagen volume fraction (r = 0.49, *p* < 0.05, Figure 6B). There was no significant correlation between M2 macrophages and collagen volume fraction (r = −0.21, *p* = 0.34, Figure 6C), although there was a trend for a negative association.

## 4. Discussion

Despite the increased morbidity and mortality associated with T2DM-induced cardiac fibrosis [8], there are no effective treatment strategies to ameliorate or reverse this fibrosis. Our study establishes for the first time that replacement of lost SP is an effective therapeutic strategy to target cardiac fibrosis in T2DM. We demonstrate that: (1) T2DM mice exhibit low circulating levels of SP concomitant with cardiac fibrosis; (2) daily replacement of SP significantly reduces cardiac fibrosis in T2DM mice; (3) SP opposes the ability of high glucose to induce cardiac fibroblast conversion to the myofibroblast pro-fibrotic phenotype, and that this involves reducing RAGE, oxidative stress and inflammatory cytokine production; and (4) SP opposes the ability of high glucose to induce a pro-inflammatory environment by promoting a beneficial shift from the pro-inflammatory M1 macrophage phenotype to the reparative M2 phenotype.

Our finding of a loss of SP in T2DM mice is in agreement with previous reports in diabetic rats [22,28] and T2DM humans [21,29]. Since SP is a sensory nerve neuropeptide, SP depletion is likely the consequence of T2DM-induced peripheral nerve degeneration, rendering nerves incapable of producing neuropeptides such as SP [30]. In previous studies [22,28], reduced SP in the diabetic rat heart corresponded with a loss of cardioprotection in response to reperfusion and post-conditioning. Replacement of SP restored cardioprotection. With this in mind, we speculated that the loss of SP in diabetes could also be a stimulus for cardiac fibrosis and hypertrophy in non-ischemic diabetic cardiomyopathy. Using the Lepr^db/db^ diabetic mouse, we established a causal relationship between the loss of SP and fibrosis by confirming that daily replacement of SP ameliorated cardiac fibrosis. This phenomenon was independent of obesity, blood glucose and blood pressure.

Seeking to identify mechanisms by which replacement SP opposed fibrosis, we firstly examined the effects of SP on isolated mouse cardiac fibroblast phenotype after exposure to high glucose. Hyperglycemic conditions have been reported to stimulate fibroblast proliferation and conversion to the myofibroblast phenotype [31,32,33,34,35,36]. In our study we observed an increase in α-SMA, as well as increased proliferative and migratory responses to high glucose, all of which are characteristic of myofibroblast conversion. SP concentration-dependently prevented these changes, indicating that the presence of SP in the culture media prevents high glucose-induced conversion to a myofibroblast phenotype. Importantly, SP was also able to significantly reduce or prevent high glucose-induced increases in ECM production by fibroblasts, including fibrillar collagens (collagen I and collagen III), as well as basement membrane ECM proteins (fibronectin and laminin). SP also reduced LOX levels that were up-regulated by high glucose. LOX is responsible for cross-linking mature collagen molecules in the ECM [37], which contributes to increased myocardial stiffness [38]. Therefore, reduced LOX indicates less collagen cross-linking in the presence of SP. Further, there was a non-significant trend for BMP-1 to be reduced by SP. BMP-1 cleaves the C-terminus of the pro-collagen molecule, the rate limiting step in the formation of collagen fibrils, ready for incorporation into the ECM [37]. Thus, in addition to reducing ECM protein production, SP also normalizes processes important to producing the mature collagen molecule and incorporating that molecule into the ECM. Overall, these data show that the absence of SP allows a pro-fibrotic fibroblast phenotype to develop in response to high glucose, whereas the presence of SP, akin to replacement SP in Lepr^db/db^ mice, opposes the effects of high glucose on fibroblast phenotype and function. Our additional finding that SP also opposed a pro-fibrotic phenotype in human cardiac fibroblasts, suggests translational relevancy for these findings.

High levels of glucose increase the synthesis of AGE’s [39]. RAGE mediates the adverse effects of AGE’s by activating multiple pathways that alter cellular function, including oxidative and nitrogen species [39]. In our study, RAGE was dramatically up-regulated on fibroblasts and concentration-dependently reduced by SP. We also observed oxidative stress in response to high glucose in the form of increased H_2_O_2_, which can result from increased superoxide [40]. SP prevented oxidative stress, although seemingly not by a mechanism involving the antioxidant superoxide dismutase. A previous study [41] identified an increase in iNOS-derived NO from cardiac fibroblasts exposed to high glucose; iNOS-derived NO contributes to oxidative stress via interactions with superoxide to form peroxinitrite [42]. We observed a trend toward increased NO production by isolated cardiac fibroblasts in response to high glucose, with high concentrations of SP further increasing NO levels. As a potent coronary vasodilator, SP is well known to cause NO production, and since NO is known to be cardioprotective [43], this raises the possibility that SP promotes a shift to so-called ‘good’ NO that then provides protective effects on cardiac fibroblasts by promoting a shift in nitric oxide synthase (NOS) isoforms from iNOS-derived ‘bad’ NO that couples with superoxide to cause oxidative stress, to eNOS and/or nNOS-derived ‘good’ NO that is cardioprotective. There is some support for this concept in cardiac microvascular endothelial cells where under high glucose conditions, SP maintained cell viability by decreasing reactive oxygen species production while elevating NO [44]. In that study, SP opposed the high glucose-induced decrease in Akt phosphorylation; Akt can regulate NOS isoforms [45,46].

Inflammatory cells such as macrophages are also involved in the pathogenesis of cardiac fibrosis [4], and macrophage phenotype is important in determining function under specific conditions. In diabetic wounds, there is persistence of M1 macrophage infiltration where this chronic inflammation impedes proper healing. Exogenous SP promotes proper wound healing and resolves the inflammatory phase in these diabetic wounds by promoting the transition of the pro-inflammatory M1 macrophages to the ‘reparative’ M2 phenotype [47,48,49]. We hypothesized that something similar could be occurring in the T2DM heart. As initial proof of concept, we cultured BMMΦ’s in high glucose and treated them with SP, utilizing TNF-α, IL-6 and CCL2 as M1 phenotype markers, and arginase-1 as well as IL-10 as M2 phenotype markers [50,51]. We found that production of pro-inflammatory M1 cytokines was up-regulated in response to high glucose; the presence of SP in the culture media prevented induction of M1 cytokines, instead promoting an M2 phenotype as indicated by increased arginase-1 and IL-10. These in vitro data served as proof of concept that the presence of SP opposes the pro-inflammatory M1 macrophage phenotype, instead favoring an M2 phenotype. Interestingly, IL-10 has been shown to oppose fibrosis in the stressed heart [52], supporting the anti-fibrotic role of M2 macrophages. Accordingly, we assessed M1 (Mac-2^+^CD86^+^) and M2 (CD206^+^) macrophages in the hearts of Lepr^db/db^ mice. In the normal mouse heart, we found only M2 macrophages, consistent with a lack of inflammation, however, there was a clear increase in M1 macrophages in untreated Lepr^db/db^ hearts, leading to a dramatic increase in the ratio of M1/M2 cells. This is consistent with other tissues in diabetes where infiltrating Ly6C^+^ monocytes differentiate into M1 macrophages with their accumulation resulting in an imbalance between M1 and M2 macrophages [53], including in a previous description in the diabetic mouse heart [54]. In our current study, replacement SP was able to shift the balance back towards a more favorable M2 phenotype, as indicated by a decreased M1/M2 ratio. Diabetic nephropathy is associated with increased M1 macrophages, while induction of M2 macrophages is associated with reduced renal damage [55]. In our study, an increased M1/M2 ratio was positively correlated with cardiac fibrosis, as was increased M1 macrophage numbers. There was a non-significant negative association between the number of M2 macrophages and fibrosis, suggesting that the ratio of M1/M2 might be what is critical, with replacement SP restoring a more favorable balance of M2 to M1 macrophages. In rat bone marrow mononuclear-GM-SCF differentiated macrophages, SP induced the M2 phenotype through direct activation of the PI3K/Akt pathway [48]. Therefore, similar to fibroblasts, we would speculate that Akt regulation of NOS isoforms may also mediate SP effects on cardiac macrophage phenotype. As an interesting side note, IL-4 is a driver of the M2 phenotype [56], and we found that SP caused cardiac fibroblasts to dramatically up-regulate IL-4 production. Thus, SP may also promote fibroblast/macrophage communication to promote an M2 phenotype.

In addition to fibrosis, diabetic cardiomyopathy also is typically considered to involve myocardial hypertrophy. We did not observe hypertrophy in our cohort of Lepr^db/db^ mice, as assessed by raw LV mass not indexed to bodyweight or tibia length. The absence of a hypertrophic response was confirmed by the lack of change in cardiomyocyte cross-sectional area in diabetic Lepr^db/db^ mice. We submit that there was a lack of hypertrophy because this cohort of Lepr^db/db^ mice were not hypertensive. Others have reported myocardial hypertrophy in Lepr^db/db^ mice at ages similar to our cohort, however, these studies also reported significantly increased blood pressure [57]. Another study reported that while there was no hypertrophy in male Lepr^db/db^ mice at 10 weeks of age [58], cardiomyocyte width was increased by 18 weeks of age, two weeks older than our mice. Interestingly though, female Lepr^db/db^ mice had developed hypertrophy at 10 weeks of age. Unfortunately, blood pressure was not reported in this study. It may be that females develop hypertrophy more rapidly in response to diabetes than males and that, unlike fibrosis, increased blood pressure rather than high glucose alone is required to induce a hypertrophic response.

**Figure 7:**
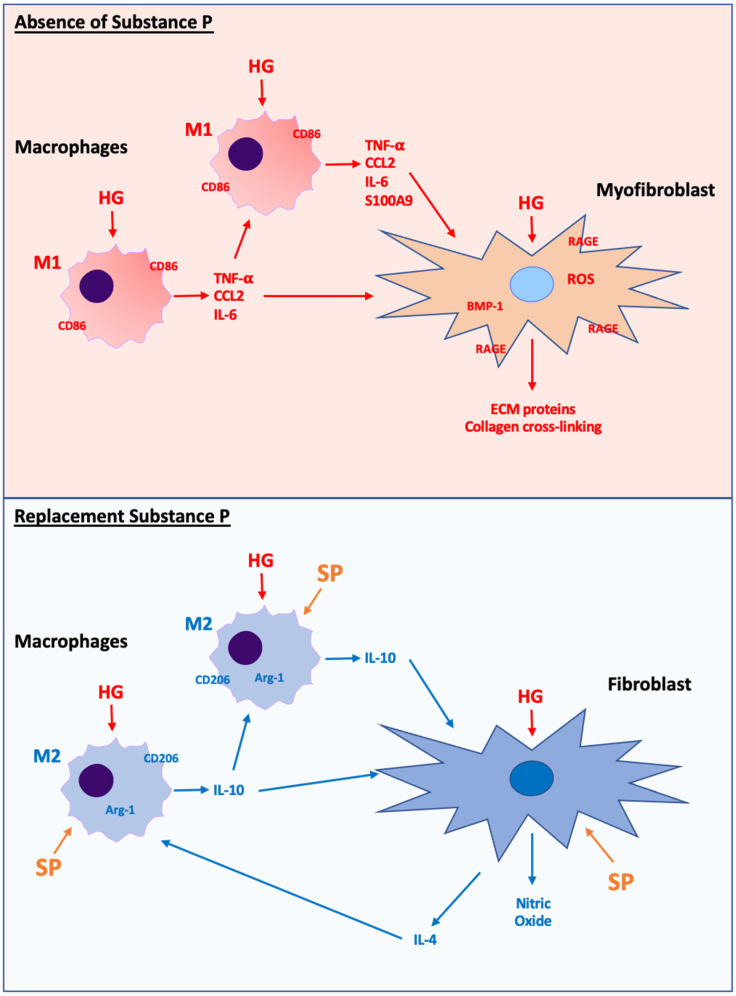
Schematic showing proposed macrophage and fibroblast phenotype responses to high glucose conditions in the absence of SP (upper panel) and high glucose conditions with replacement SP (lower panel). Under conditions of high glucose in the absence of SP, macrophages take on a pro-inflammatory M1 phenotype that releases pro-inflammatory cytokines (TNF-α, CCL2, IL-6), which can act on fibroblasts to promote a pro-fibrotic phenotype by overwhelming reparative M2 macrophages. High glucose in the absence of SP also acts directly at the fibroblast to promote a pro-fibrotic phenotype mediated by increased RAGE, oxidative stress, and inflammatory cytokines (TNF-α). Conversely, when SP is present in addition to high glucose, M1 macrophages are reduced and M2 macrophages become the dominant macrophage phenotype. M2 macrophages release the anti-fibrotic cytokine IL-10. Additionally, the presence of SP opposes the pro-fibrotic fibroblast phenotype by reducing RAGE and oxidative stress, as well as increasing NO production. In response to SP, cardiac fibroblasts also produce IL-4, which can act as an additional stimulus for M2 macrophage development.

In summary, we report a loss of SP in T2DM mice, and that this loss of SP predisposes the diabetic heart to developing fibrosis, since cardiac fibrosis could be ameliorated by replacement of SP. The protective actions of SP involve manipulation of cardiac fibroblasts, opposing high glucose induction of a pro-fibrotic fibroblast phenotype. This involves down-regulation of RAGE, oxidative stress and inflammation, as well as up-regulation of NO as depicted in Figure 7. Replacement SP also promotes a shift in macrophage phenotype from the pro-inflammatory M1 phenotype to the reparative M2 phenotype (Figure 7). Thus, this study identifies replacement SP as a potential treatment for diabetic cardiac fibrosis. However, this study does have limitations. We did not examine the actions of SP on the cellular effects of increased fatty acids. In addition to the adverse effects of hyperglycemia in the diabetic state, dyslipidemia also contributes to cellular dysfunction, including pathways that regulate oxidative stress [59,60]. SP has been shown to decrease fatty acid uptake and lipid droplet accumulation in 3T3-L1 adipocytes [61]. In adipose tissue, SP blocked insulin-mediated stimulation of fatty acid uptake [61]. Therefore, some of the effects of SP on fibroblast and even macrophage phenotype and function in our study could have involved modulation of fatty acid cellular effects. A further limitation is that a mouse model is not ideal for identifying possible side effects of replacement SP therapy. Having said that, we did not observe any obvious off-target effects of treatment. That a loss of SP occurs in the diabetic human heart [21], together with our finding that SP opposes the pro-fibrotic actions of high glucose on human cardiac fibroblasts, indicates the translational significance of our study and the need to pursue further studies.

## Data Availability

The data underlying this article will be shared on reasonable request to the corresponding author.
